# Automated data collection tool for real-world cohort studies of chronic hepatitis B: Leveraging OCR and NLP technologies for improved efficiency

**DOI:** 10.1016/j.nmni.2024.101469

**Published:** 2024-08-28

**Authors:** Xiaomei Zhou, Tao Zeng, Yibo Zhang, Yingying Liao, Jaime Smith, Lin Zhang, Chao Wang, Qinghai Li, Dongbo Wu, Yutian Chong, Xinhua Li

**Affiliations:** a Information Center, The Third Affiliated Hospital of Sun Yat-sen University, Guangzhou, 510630, China; b Department of Infectious Diseases, The Third Affiliated Hospital of Sun Yat-sen University, Guangdong Key Laboratory of Liver Disease, Guangzhou, 510630, China; c PAREXEL International, Billerica, MA, 01821, USA; d PAREXEL International, Shanghai, 200120, China; e KingPoint Data Technology Co., Ltd, Guangzhou, 510635, China; f West China Hospital of Sichuan University, Chengdu, 610041, China

**Keywords:** Data collection, Hepatitis B, Natural language processing, Optical character recognition

## Abstract

**Background:**

Collecting and standardizing clinical research data is a very tedious task. This study is to develop an intelligent data collection tool, named CHB-EDC, for real-world cohort studies of chronic hepatitis B (CHB), which can assist in standardized and efficient data collection.

**Methods:**

CHB_EDC is capable of automatically processing various formats of data, including raw data in image format, using internationally recognized data standards, OCR, and NLP models. It can automatically populate the data into eCRFs designed in the REDCap system, supporting the integration of patient data from electronic medical record systems through commonly used web application interfaces. This tool enables intelligent extraction and aggregation of data, as well as secure and anonymous data sharing.

**Results:**

For non-electronic data collection, the average accuracy of manual collection was 98.65 %, with an average time of 63.64 min to collect information for one patient. The average accuracy CHB-EDC was 98.66 %, with an average time of 3.57 min to collect information for one patient. In the same data collection task, CHB-EDC achieved a comparable average accuracy to manual collection. However, in terms of time, CHB-EDC significantly outperformed manual collection (p < 0.05). Our research has significantly reduced the required collection time and lowered the cost of data collection while ensuring accuracy.

**Conclusion:**

The tool has significantly improved the efficiency of data collection while ensuring accuracy, enabling standardized collection of real-world data.

## 1 Introduction

Hepatitis B virus (HBV) infection is a global epidemic. According to the World Health Organization (WHO), the global prevalence of HBsAg in the general population is 3.8 %, with approximately 1.5 million new cases of HBV infection, 296 million cases of chronic infection, and 820,000 deaths due to HBV-related liver failure, cirrhosis, or hepatocellular carcinoma (HCC) in 2019 [1]. China has the highest number of HBV-infected individuals, accounting for one-third of the world's infected population, posing a serious threat to patients' lives and imposing a heavy burden on the healthcare system [2]. To achieve the World Health Organization's goal of “eliminating viral hepatitis as a public health threat by 2030,” large-scale, multicenter studies on the clinical research of chronic HBV infection are essential.

In real-world cohort studies of chronic hepatitis B (CHB), structured patient data serves as the foundation for large-scale, multicenter analysis of real-world HBV cohort data, and it has significant clinical significance for the comprehensive prevention and treatment of hepatitis B. However, the traditional manual data collection is prone to human errors, and the quality and reliability of the data are insufficient, resulting in low efficiency and inability to meet the requirements of large-scale multicenter real world cohort studies. This challenge is mainly manifested in two aspects: (1) there is a large amount of unstructured data in electronic medical records [3], such as laboratory test reports, discharge summaries, and chief complaint, current medical history, past history, personal history, family history, and allergy history; (2) in multicenter real-world studies, there are differences in data among different centers, with some centers storing data in the form of images or paper documents, while others use electronic spreadsheet databases. To address this challenge, mature artificial intelligence (AI) technologies in the field of computer science, such as Optical Character Recognition (OCR) [4] and Natural Language Processing (NLP) [5,6], can be used for automated data extraction. These technologies offer intelligent proofreading functions, precise and efficient data collection, and significant cost reduction, providing guarantees for large-scale, multicenter data collection. OCR technology can recognize and extract text information from images and scanned documents, thereby achieving electronic data collection, and NLP technology can extract key information from unstructured data and convert it into structured data [4]. The application of AI technologies such as NLP and OCR in research data collection can significantly reduce the manual cost of data collection, cleaning, transformation, and standardization, while improving accuracy and efficiency [5].

**Fig. 1:**
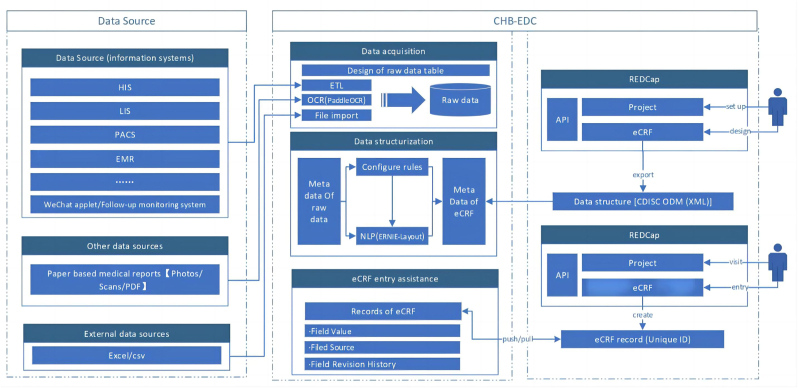
CHB-EDC system design diagram. CHB-EDC supports data collection from various sources. HIS, hospital information system; LIS, laboratory information management system; PACS, picture archiving and communication system; EMR, electronic medical record system.

In this study, we developed an AI-assisted data collection tool, named Chronic Hepatitis B Electronic Data Capture (CHB-EDC) System, for real-world data collection in the CHB research cohort as shown in Fig. 1. This tool can automatically process various formats of data, including raw data in image format, using internationally recognized data standards, OCR, and NLP models. It can automatically fill in the electronic case report forms (eCRFs) designed in the Research Electronic Data Capture (REDCap) system with data from image and other format sources [7]. Additionally, it intelligently extracts, summarizes, de-identifies, and facilitates data sharing, markedly enhancing the precision and efficiency of research data collection.

## 2 Methods

### 2.1 Requirements and technology selection

CHB-EDC system was designed in strict accordance with the “Implementation Specification for Real-World Research in Clinical Medicine in China” [8]. At the same time, the data definition and processing standards were referenced from the Clinical Data Interchange Standards Consortium (CDISC) standards to meet the requirements of multicenter research both domestically and internationally [9]. As shown in Fig. 1, our application development is divided into 4 stages. In the first stage, OCR and NLP tools were developed based on artificial intelligence technology. In the second stage, these tools were integrated into the REDCap system to achieve automated mapping of data into eCRF forms. In the third stage, integration with the electronic medical record (EMR) system was achieved through interfaces, enabling standardized data collection for both internal and external research purposes. In the fourth stage, we deployed our tool to the collaborating hospitals and validation.

### 2.2 Stage 1: research preparation and OCR/NLP models development

The study subjects must meet the definition of chronic hepatitis B prevention and treatment guidelines in China (2019 edition) [10] and also meet the following criteria. Inclusion criteria: (1) The time interval between the first positive Hepatitis B Surface Antigen (HBsAg) test and the last test is greater than 6 months; (2) The current examination data of the patients show normal Alanine Aminotransferase (ALT) levels; (3) Liver biopsy has been performed and corresponding pathological data are available; (4) Patients have not received antiviral treatment (including nucleoside (acid) or interferon) before liver biopsy. Exclusion criteria: (1) Patients with overlapping infections of Hepatitis A Virus (HAV), Hepatitis C Virus (HCV), Hepatitis D Virus (HDV), Hepatitis E Virus (HEV), or Human Immunodeficiency Virus (HIV); (2) Patients with other liver diseases (such as drug-induced, alcoholic, autoimmune, genetic metabolic liver diseases, etc.); (3) Patients who have undergone organ transplantation or are preparing for organ transplantation; (4) Other situations deemed unsuitable for inclusion by the researchers. Experts in liver disease research discussed and formulated the fields and format of the eCRF. The planned collection of patient information includes demographic information, vital signs, complete blood count, liver function, hepatitis B virus markers, and pathology reports. The study subjects had to meet the definition of chronic hepatitis B outlined in the 2019 Chinese Guidelines for the Prevention and Treatment of Chronic Hepatitis B [11].

**Fig. 2:**
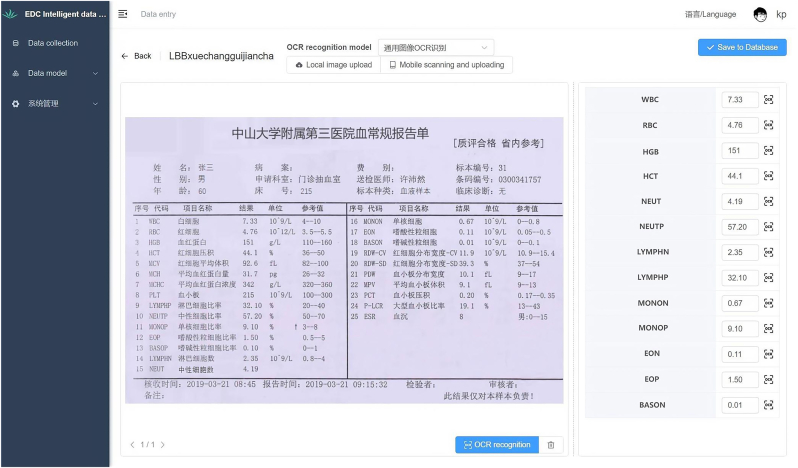
OCR tool text recognition interface. This tool supports the digitization and collection of data from paper documents, images, and scanned files.

**Fig. 3:**
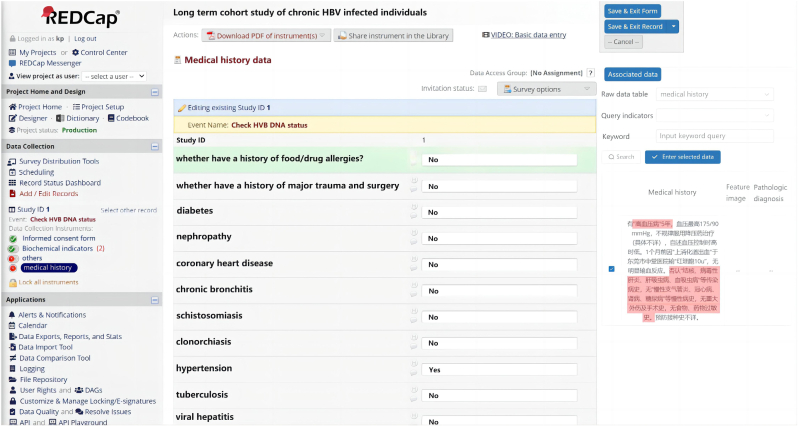
NLP tool data structuring interface. This tool can convert unstructured Chinese text into structured data and supports switching between Chinese and English.

In order to digitize a large amount of external paper-based and image-format patient data, we utilized OCR technology. PaddleOCR is an open-source OCR tool developed based on Baidu's PaddlePaddle deep learning platform [12]. It supports accurate recognition of various types of text, including printed text, handwritten text, and scene text (Fig. 2). NLP models have been widely applied in the processing of clinical semi-structured and unstructured data. We used the ERNIE-Layout model for understanding and transforming clinical semi-structured and unstructured data [13]. ERNIE-Layout is an open-source model for cross-modal document understanding. It utilizes layout knowledge enhancement technology to integrate text, images, and layout information for joint modeling, enabling deep understanding and analysis of multi-modal documents such as document images and scans (Fig. 3). Our model's pretraining data was obtained from the Chinese Medical Entity Extraction dataset (CMeEE), which is part of the Chinese Biomedical Language Understanding Evaluation (CBLUE) benchmark 3.0 version. It consists of 15000 training data, 5000 validation data, and 3000 test data for Chinese medical named entity recognition.

### 2.3 Stage 2: integrated into REDCap

**Fig. 4:**
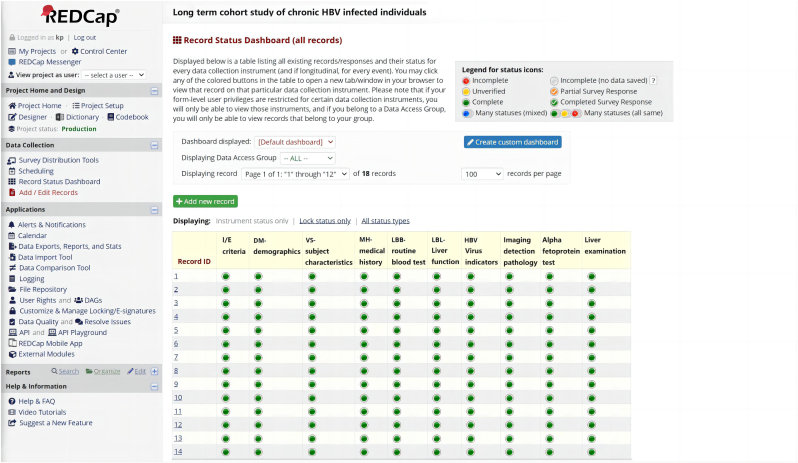
Data collected by AI tools automatically mapped to eCRF in REDCap according to the set rules.

The next stage involved developing server programming to automatically extract data from OCR and NLP models and populate the electronic case report forms (eCRF) designed in the Research Electronic Data Capture (REDCap) system (Fig. 4). To ensure the security and privacy of patient data, CHB-EDC implemented strict user access restrictions and maintained an activity log tracking feature. To address security concerns during the data storage and sharing process, we designed and developed a data security protection tool. This tool is a web-based system with a B/S architecture that integrates methods such as data encryption and de-identification. It allows for encryption [14] and de-identification [15] of structured data, ensuring that locally sensitive files are uploaded in an encrypted form and stored in the database. During data exchange, the tool ensures that the data remains in a de-identified and encrypted state. Only after completing the data exchange can the data be decrypted, which ensures a comprehensive data protection process. CHB-EDC utilizes Node.js as the server platform, enabling seamless code reuse between the browser and server using JavaScript with minimal editing. This stage took place from June to August 2023, during which the application underwent further testing for browser compatibility.

### 2.4 Stage 3: integrated into EMR

The third stage involved integrating the CHB-EDC system into the hospital's EMR system. This integration was achieved through a data interface, which allowed for automatic collection of patient data from within the hospital to CHB-EDC using a single HTTPS interface. The application would periodically collect data from the EMR system for patients with chronic hepatitis B. Using the eCRF data format, the ERNIE-Layout model was employed to process unstructured and semi-structured data, automatically populating the eCRF in the REDCap system. This significantly reduced the workload for clinicians in collecting research data. For patient records brought from other medical institutions in the form of photocopies or images, the PaddleOCR model was used to digitize them, and then the ERNIE-Layout model was applied to map the data to the eCRF.

### 2.5 Stage 4: deployment and validation

Our CHB-EDC tool has been deployed at the Third Affiliated Hospital of Sun Yat-sen University and West China Hospital of Sichuan University. This study has collected clinical pathological and virological records of 4072 patients from the Third Affiliated Hospital of Sun Yat-sen University and West China Hospital of Sichuan University for evaluating the performance of our CHB-EDC tool in data collection. Four research assistants were recruited to annotate the data, and their analysis conclusions were compared. If there were significant differences in the analysis conclusions, a senior expert physician was invited to conduct further analysis, and the conclusions of the senior expert were considered final.

The evaluation metrics in this study included the average accuracy of data collection for paper medical records (calculated per field based on the eCRF design in this study), and the average time taken per record (in minutes), independent two-sample *t*-test was performed. The records were categorized into demographic information, vital signs, complete blood count, liver function, hepatitis B virus indicators, and pathological reports, totaling 6 categories.

## 3 Results

We conducted 10 experiments from October to December 2023, with the experimental group using CHB_EDC for data collection and the control group consisting of four test subjects for manual collection. Each experiment involved randomly sampling 100 paper medical records and comparing the accuracy and time taken for field recognition between manual collection and CHB_EDC collection. The experimental results showed that the average accuracy of manual collection was 98.65 %, with an average time of 63.64 min to collect information for one patient. The average accuracy of CHB_EDC collection was 98.66 %, with an average time of 3.57 min to collect information for one patient, as shown in Table 1. It is noteworthy that the time required for data collection using CHB_EDC consists of two parts: one part is the time used for model inference, and the other part is for manual verification of data quality and error correction. CHB_EDC supports highlighting the positions where field boxes are empty and their corresponding text, assisting staff in quickly verifying them. In the same data collection task, CHB_EDC achieved a comparable average accuracy to manual collection. However, in terms of time, CHB_EDC significantly outperformed manual collection (p < 0.05), saving an average of 60.07 min per medical record data collection. CHB_EDC, built upon the REDCap tool, is an OCR and NLP based tool specifically designed for non-electronic and non-structured data. It inherits the capabilities of data digitization and structured processing, greatly reducing the cost of data collection and preprocessing.

**Table 1:** Comparison of Accuracy between manual collection and CHB_EDC collection verification. SD: standard deviation.

Data Types	Manual model	CHB_EDC facilitated manual model
	Mean accuracy (SD) %	Mean accuracy (SD) %
Demographic information	98.99(0.98)	98.70(0.86)
Vital signs	98.64(0.90)	98.51(0.92)
Complete blood count	98.68(0.71)	98.61(0.78)
Liver function	98.53(1.06)	98.67(1.00)
Hepatitis B virus markers	98.80(0.85)	98.74(0.92
Pathological reports	98.28(1.09)	98.72(0.90)
Total	98.65(0.95)	98.66(0.90)

## 4 Discussion

This research is the first to develop a customized tool for the collection and standardization of clinical data for chronic hepatitis B. Previous research has applied OCR and NLP technologies to medical data processing with good results. Building on prior work, we evaluated the practical needs of collecting and standardizing clinical data for chronic hepatitis B, considering algorithm complexity (computational requirements), development challenges, cost, and efficiency. As a result, we developed an economically practical tool for data collection and structuring in chronic hepatitis B, known as CHB-EDC.

AI holds tremendous potential in the field of liver disease research, but currently, it is primarily focused on disease diagnosis and prognosis prediction [16,17]. Most studies do not address issues related to data collection and organization [18]. These predictive models require substantial manual data standardization from the modeling stage to the application and inference stage, which is not conducive to the subsequent research and use of the models.

In clinical research on chronic hepatitis B, our CHB-EDC tool serves as an auxiliary tool for early-stage data collection and standardization. Considering requirements such as cost and accuracy, we have chosen to optimize existing mature, open-source OCR and NLP models. Medical large language models have great potential in the field of clinical text data standardization processing. However, these models have a very large number of parameters and have not yet been adapted for the application of data collection and standardization in the context of chronic hepatitis B. The cost of development and training is very high, so large language models are not being used. For example, Med-PaLM performs exceptionally well in answering medical questions [19], PubMedGPT incorporates millions of full-text articles and abstracts in its training to interpret biomedical research language and assist in medical research [20]. GatorTron, developed by the University of Florida, is an electronic health record (EHR) big data model with 8.9 billion parameters, although much smaller than Med-PaLM. Currently, the application of large language models in medicine is still being explored and does not yet support specific fields, such as the application of data collection and standardization for chronic hepatitis B [21]. The high development and maintenance costs are also difficult for the majority of medical research teams to bear, so we need to choose traditional NLP solutions. The traditional NLP models include the BERT model and its variant models, with ERNIE-Layout being one of them. ERNIE-Layout incorporates a large amount of Chinese information material in the training corpus, and we only need to fine-tune it to achieve the structured and standardized clinical text data for chronic hepatitis B. The OCR model in CHB-EDC is used for the electronic collection of printed data from patient paper medical records, examination reports, and other documents. The features of these documents are white background with black text, and they hardly present challenges such as blurry images, complex backgrounds, messy handwriting, or complex semantic characters, which are common in cutting-edge OCR research [22]. Therefore, the recognition difficulty is not significant. Lianchi Zheng et al. compared the performance of Bert without additional coordinate information and LayoutXLM with additional coordinate information to improve medical OCR information extraction [23]. The open-source PaddleOCR model has achieved an accuracy rate of 99.5 %, and it can be directly invoked without the need for re-modeling and retraining.

In the management of hepatitis B patients, timely and accurate data is crucial for developing appropriate treatment plans. We have developed the CHB-EDC tool, which incorporates OCR and NLP technologies to enable the digitization and structured extraction of paper medical records and laboratory reports. This greatly simplifies the data collection and organization process. Traditional manual data collection requires personnel to enter, modify, and verify each data point, while CHB_EDC only requires partial manual data collection for model training and optimization. This allows for the creation of a pipeline for batch processing, significantly reducing the time required for data collection. Compared to traditional manual data collection, the average time for CHB_EDC data collection and verification is 3.57 min, which is 17.83 times faster than the traditional manual collection method, which takes an average of 63.64 min. Once successfully deployed, CHB_EDC can be reused multiple times, supporting batch processing for large-scale multicenter data collection needs. This significantly reduces time costs and lowers the difficulty of data collection in clinical research involving a large number of multicenter patient data.

In terms of accuracy, we compared the error rates of six different data types and found that when CHB_EDC data collection was combined with manual review, the accuracy was comparable to manual data collection. This indicates that CHB_EDC can achieve an average accuracy rate of 98.66 %, which is comparable to the accuracy of manual data collection at 98.65 %. This offers the potential to replace traditional manual operations and reduce repetitive labor for researchers. During the modeling and validation process, we found some methods that could improve accuracy. For example, (1) incorporating synonym mapping to address the issue of slight variations in vocabulary used in medical records from different hospitals, (2) including data from multiple hospitals in the training dataset to prevent potential overfitting issues from single-center data, and (3) supplementing with manual review for data collection, which can assist in data quality audit by highlighting text and field boxes. In this study, a total of 4072 medical records from Sun Yat-sen University Third Affiliated Hospital and Sichuan University West China Hospital were included for modeling and validation testing. The CHB-EDC tool effectively supported data collection for chronic hepatitis B patients from both hospitals.

On the other hand, by digitizing paper-based data through OCR technology, we preserve the integrity of the original data and use NLP technology to structure unstructured data, making it easier to manage and trace. This provides a reliable data foundation for long-term follow-up and treatment evaluation of hepatitis B patients. To protect sensitive data involving patient privacy, we have introduced encryption and de-identification algorithms to defend against potential privacy and security risks, ensuring that patient information is not misused.

However, this study also has certain limitations. Firstly, the use of AI technologies, such as OCR and NLP, requires high hardware and software requirements during model training and optimization. The technical capabilities and equipment differences among different medical institutions may affect the generalization and application of the methods. Additionally, although OCR and NLP technologies have made significant progress, there are still error rates when dealing with complex and diverse data in the medical field. Specifically, when processing unstructured data, the model's adaptability to specific domains needs further optimization. Next, we plan to optimize the performance and expand the functionality of our data collection tool from two aspects. Firstly, we have signed contracts with eight hospitals in China, and CHB-EDC will collect 200 medical records from each of these hospitals, totaling 1600 medical records for model optimization and validation. Finally, CHB-EDC will be deployed in the partner hospitals for subsequent joint research project data collection. On the other hand, we will use the same technical approach to develop an intelligent data collection tool for hepatitis B-related liver cirrhosis and liver cancer, in response to the research team's demand for data collection on other liver diseases. We have uploaded the project code to the GitHub open-source community (https://github.com/Clara10086/CHB-EDC) for reference by future researchers and staff involved in chronic hepatitis B data collection.

## 5 Conclusion

Based on the Chinese Chronic Hepatitis B Prevention and Treatment Expert Guidelines and the international CDISC data standards, we have developed a CHB-EDC system that incorporates OCR and NLP technologies to expand the functionality of the REDCap non-electronic data and non-structured data collection, transformation, and management tool. CHB-EDC is capable of automatically processing various formats of data, including raw data in image format, through CDISC standards, OCR, and NLP models. It can automatically populate data from image and other format source data into eCRF, while the system also includes built-in de-identification and encryption tools to protect data privacy and security. In the context of a large number of hepatitis B patients and complex disease information, the introduction of artificial intelligence technology makes it more efficient, secure, and accurate to extract key information from a large amount of unstructured data. This study provides an innovative means of data collection and processing for hepatitis B patient management and related research, offering a feasible solution for multicenter data integration and serving as a reference for other chronic disease research.

## Ethics statement

This study was supported by the 5010 Cultivation Program of Clinical Research of Sun 10.13039/100022952Yat-Sen University (2018024), Guangdong Province Rural Science and Technology Assistance (Special Commissioner) Project（No. KTPYJ2022012).

## CRediT authorship contribution statement

**Xiaomei Zhou:** Writing – original draft, Investigation, Formal analysis. **Tao Zeng:** Writing – original draft. **Yibo Zhang:** Writing – original draft. **Yingying Liao:** Methodology. **Jaime Smith:** Methodology. **Lin Zhang:** Software, Formal analysis. **Chao Wang:** Software. **Qinghai Li:** Methodology. **Dongbo Wu:** Conceptualization. **Yutian Chong:** Conceptualization. **Xinhua Li:** Project administration, Conceptualization.

## Declaration of competing interest

The authors of this article entitled “Automated Data Collection Tool for Real-World Cohort Studies of Chronic Hepatitis B: Leveraging OCR and NLP Technologies for Improved Efficiency” declare that they have no conflicts of interest to disclose.
